# The Olive Biophenols Oleuropein and Hydroxytyrosol Selectively Reduce Proliferation, Influence the Cell Cycle, and Induce Apoptosis in Pancreatic Cancer Cells

**DOI:** 10.3390/ijms19071937

**Published:** 2018-07-02

**Authors:** Chloe D. Goldsmith, Danielle R. Bond, Helen Jankowski, Judith Weidenhofer, Costas E. Stathopoulos, Paul D. Roach, Christopher J. Scarlett

**Affiliations:** 1 Pancreatic Cancer Research Group, School of Environmental & Life Sciences, University of Newcastle, Ourimbah 2258, NSW, Australia; Danielle.Bond@newcastle.edu.au (D.R.B.); C.Scarlett@newcastle.edu.au (C.J.S.); 2 Faculty of Science, The University of Newcastle, Ourimbah 2258, NSW, Australia; Paul.Roach@newcastle.edu.au; 3 Faculty of Health, The University of Newcastle, Ourimbah 2258, NSW, Australia; Helen.Jankowski@uon.edu.au (H.J.); Judith.Weidenhofer@newcastle.edu.au (J.W.); 4 School of Science, Engineering and Technology, University of Abertay, Dundee, Scotland DD1 1HG, UK; c.stathopoulos@abertay.ac.uk; 5 Hunter Medical Research Institute (HMRI), New Lambton Heights 2305, NSW, Australia

**Keywords:** olive, phenolic compound, nutraceutical, chemoprevention, anti-cancer, MIA PaCa-2, HPDE, oleuropein, hydroxytyrosol

## Abstract

Current chemotherapy drugs for pancreatic cancer only offer an increase in survival of up to six months. Additionally, they are highly toxic to normal tissues, drastically affecting the quality of life of patients. Therefore, the search for novel agents, which induce apoptosis in cancer cells while displaying limited toxicity towards normal cells, is paramount. The olive biophenols, oleuropein, hydroxytyrosol and tyrosol, have displayed cytotoxicity towards cancer cells without affecting non-tumorigenic cells in cancers of the breast and prostate. However, their activity in pancreatic cancer has not been investigated. Therefore, the aim of this study was to determine the anti-pancreatic cancer potential of oleuropein, hydroxytyrosol and tyrosol. Pancreatic cancer cells (MIA PaCa-2, BxPC-3, and CFPAC-1) and non-tumorigenic pancreas cells (HPDE) were treated with oleuropein, hydroxytyrosol and tyrosol to determine their effect on cell viability. Oleuropein displayed selective toxicity towards MIA PaCa-2 cells and hydroxytyrosol towards MIA PaCa-2 and HPDE cells. Subsequent analysis of Bcl-2 family proteins and caspase 3/7 activation determined that oleuropein and hydroxytyrosol induced apoptosis in MIA PaCa-2 cells, while oleuropein displayed a protective effect on HPDE cells. Gene expression analysis revealed putative mechanisms of action, which suggested that c-Jun and c-Fos are involved in oleuropein and hydroxytyrosol induced apoptosis of MIA PaCa-2 cells.

## 1. Introduction

Adherence to a Mediterranean diet is associated with a reduced risk for heart disease and most cancers, including pancreatic cancer [1,2,3,4]. One of the major differences between Mediterranean diets and other healthy diets is the high intake of olives and olive oil; the annual intake of olive oil in Mediterranean countries can range 15.3–23 kg per capita [5,6]. Many of the health benefits associated with consuming olive oil have been attributed to its high concentration of biophenols [5].

Oleuropein is a major biophenol for olive products (olives, olive oil and olive leaves). However, oleuropein is often degraded during the processing of olives to olive oil, hence, the concentration of oleuropein in extracts from olive products will vary. Regardless, oleuropein can reach up to 14% of the dry weight of olives and olive oil can contain up to 2.8 mg/kg. In addition, olive leaf extracts can contain up to 61.56 g/kg [7,8,9,10,11,12].

**Figure 1:**
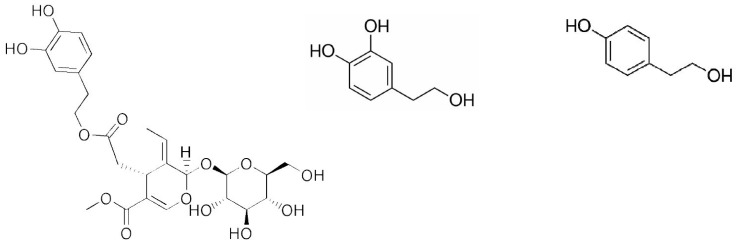
Structures of: oleuropein (**left**); hydroxytyrosol (**middle**); and tyrosol (**right**).

Hydroxytyrosol is one of the most potent antioxidants in olive oil. Hydroxytyrosol and tyrosol are degradation products of oleuropein both in the olive fruit and in the body (Figure 1). Hydrolysis of oleuropein occurs in the fruit during maturation and processing [13] and after ingestion of oleuropein, it is broken down by lipase activity and converted to hydroxytyrosol [14]. The hydroxytyrosol and tyrosol content of olive products can also vary. Interestingly, the hydrolysis of oleuropein during olive oil processing often results in higher concentrations of hydroxytyrosol present in olive oil compared to olives [7,15].

The activity of oleuropein and hydroxytyrosol in vitro has been well characterised for certain cancers. For example, oleuropein has induced apoptosis in colon cancer and breast cancer cells via activation of the p53 pathway [16,17]. Additionally, oleuropein inhibited the proliferation and induced thiol modifications, γ-glutamylcysteine synthetase and reactive oxygen species in prostate cancer cells [18]. Oleuropein has also activated apoptosis in hepatocellular carcinoma cells by suppression of the phosphatidylinositol 3-kinase/protein kinase B pathway [19]. In addition, hydroxytyrosol has demonstrated a range of activities in vitro. Hydroxytyrosol treatment of leukaemia cells resulted in the induction of apoptosis while not affecting primary human cells, including lymphocytes and polymorphonuclear cells [20], highlighting the chemo-preventative potential of hydroxytyrosol. Hydroxytyrosol treatment of vascular endothelial cells resulted in an upregulation of PI3K/Akt and Erk 1/2 and the subsequent activation of the Nrf-2 pathway exhibiting cardio-protective effects [21].

Oleuropein and hydroxytyrosol have also exhibited anti-cancer activity in vivo. Oleuropein treatment in combination with doxorubicin significantly reduced tumour volume of breast cancer xenografts [22] and melanoma tumour volumes [23] in mice and prevented ultraviolet B radiation-induced carcinogenesis in nude mice at a dose of 25 mg/kg/day [24]. Hydroxytyrosol exhibited anti-inflammatory properties in mice at a dose of 5 mg/kg [25] and inhibited the growth of cholangiocarcinoma xenografts in mice after a dose of 250 mg/kg/day [26]. Moreover, a dose of 20 mg/kg of hydroxytyrosol significantly inhibited tumour growth, angiogenesis and the activation of the AKT and NF-κB pathways in an orthotopic model of human hepatocellular carcinoma in nude mice [27].

Pancreatic cancer is a devastating disease with a five-year survival rate of less that 8%. Resistance to conventional treatment options and the toxicity of current chemotherapy agents, such as gemcitabine, makes pancreatic cancer a vital target for the development of novel therapeutic agents [28]. While oleuropein and hydroxytyrosol have been explored as anti-cancer agents for different tissues, their activity in pancreatic cancer has yet not been determined. Considering the link between adherence to a Mediterranean diet and reduced pancreatic cancer risk [29], the anti-pancreatic cancer potential of oleuropein and hydroxytyrosol warrants investigation.

We have previously shown that olive leaf extracts, containing high concentrations of oleuropein, reduced the viability of pancreatic cancer cells (MIA PaCa2) in a dose dependant manner [10]. However, crude extracts are a complex mixture of compounds and it was not possible to determine the compound/s responsible for the observed effects. Considering the desperate need for new treatments for pancreatic cancer, the potential exhibited by olive biophenols and our previous observations, the aim of this study was to investigate the effects of the major olive biophenols, oleuropein and hydroxytyrosol, on pancreatic cancer cells in vitro.

## 2. Results

### 2.1. Treatment with Olive Biophenols Reduces Pancreatic Cancer Cell Viability

The viability of pancreas cancer (MIA PaCa-2, BxPC-3 and CFPAC-1) and non-tumorigenic pancreas (HPDE) cells treated with oleuropein, hydroxytyrosol or tyrosol was assessed using a CCK8 viability assay to determine effective doses for each drug. Neither oleuropein nor hydroxytyrosol had any effect on the viability of BxPC-3 or CFPAC-1 cells (Table 1) in the treatment range tested (0–300 µM). However, both compounds dose dependently inhibited the proliferation of MIA PaCa-2 cells; the IC_50_ for oleuropein and hydroxytyrosol were 150.1 µM and 75.1 µM, respectively (Table 1).

In contrast to its effects on MIA PaCa-2 cells, oleuropein did not reduce the viability of HPDE cells, even at the concentration of 300 µM (Table 1). Considering the usual sensitivity of HPDE cells to cytotoxic drugs (Gemcitabine, IC_50_ = 0.04 nM), the activity of oleuropein and hydroxytyrosol was investigated further. However, tyrosol did not have any influence on the viability of any of the pancreatic cells within the treatment range tested (Table 1) and, hence, the activity of tyrosol was not explored further.

### 2.2. Oleuropein and Hydroxytyrosol Induce Morphological Changes in Pancreatic Cells

**Figure 2:**
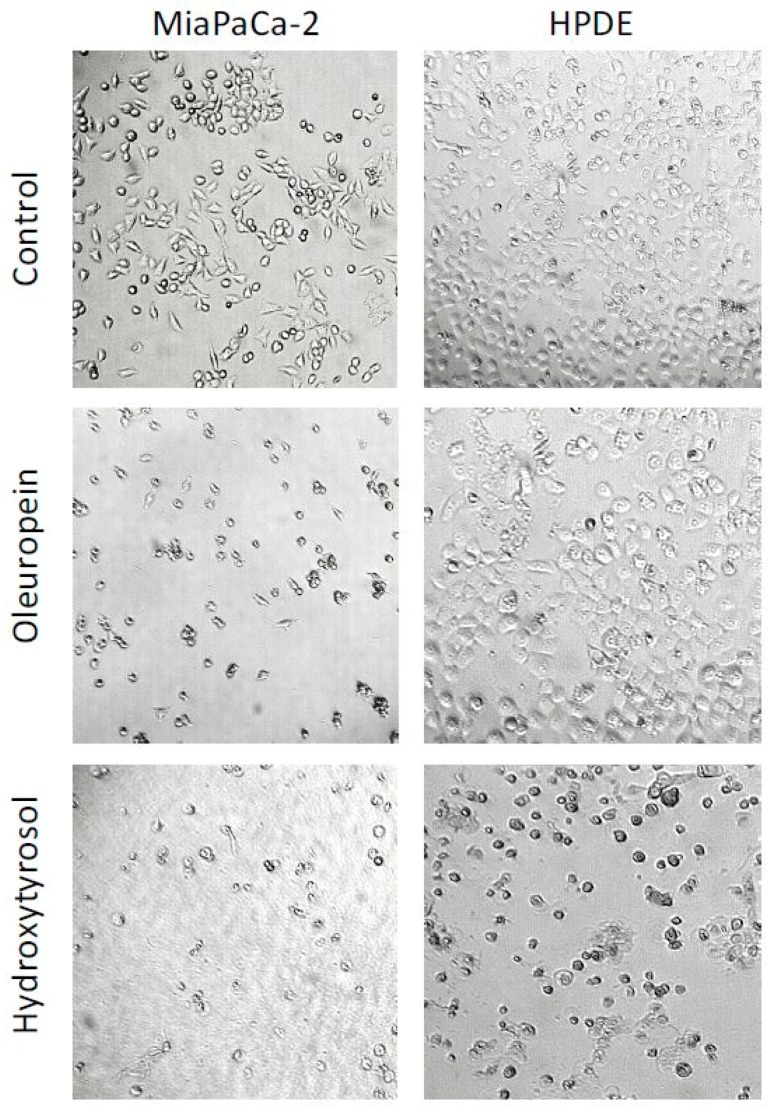
Morphological changes of MIA PaCa-2 and HPDE cells when treated with oleuropein and hydroxytyrosol for 24 h at ×100 magnification.

Significant morphological changes were observed following treatment with oleuropein and hydroxytyrosol (Figure 2). Cell shrinkage and the formation of apoptotic bodies were identified in MIA PaCa-2 cells treated with oleuropein or hydroxytyrosol and hydroxytyrosol treatment of HPDE cells also caused similar effects. Importantly, oleuropein did not induce any morphological changes in HPDE cells when compared to vehicle control (Figure 2).

### 2.3. Olive Biophenols Cause G2/M Cell Cycle Arrest in Pancreatic Cells

**Figure 3:**
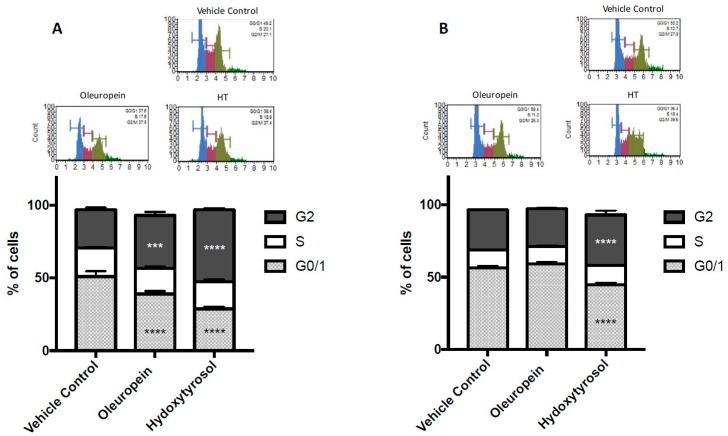
Cell cycle analysis of MIA PaCa-2 (**A**) and HPDE (**B**) cells treated with oleuropein (200 µM) and hydroxytyrosol (100 µM) for 24 h. Bar graphs show the percentage of cells in G0/1, S and G2 phase of the cell cycle measured by MUSE cell cycle analysis kit. A representative DNA content profile for vehicle control, oleuropein and hydroxytyrosol (HT) treatment is pictured for MIA PaCa-2 (**A**) and HPDE (**B**) cells. Ordinary two-way ANOVA and Tukey’s multiple comparisons test compare the percentage of treated cells (oleuropein or hydroxytyrosol) in each stage of the cell cycle to vehicle control. **** *p* < 0.0001.

The cell cycle is one of the first cell regulatory mechanisms that can be affected prior to apoptosis. Therefore, propidium iodide staining and subsequent MUSE flow cytometry analysis determined the effect of oleuropein and hydroxytyrosol on the cell cycle. Treatment of MIA PaCa-2 cells with oleuropein or hydroxytyrosol caused cell cycle arrest at the G2 phase (Figure 3A); there was a significant increase in the percentage of cells in G2 (10.1% and 23.1% increase, *p* < 0.0001 and <0.0001, respectively), coupled with a decrease in the percentage of cells in G0/1 (11.9% and 22.3% decrease, *p* < 0.0001 and <0.0001, respectively) compared to vehicle control (Figure 3A).

In HPDE cells, oleuropein did not have a significant effect (Figure 3B) on the number of cells in G0/1 or G2 phase (*p* = 0.058 and 0.3088, respectively). However, hydroxytyrosol treatment of HPDE cells caused a significant increase in in the percentage of cells in G2 (7.3% increase, *p* < 0.0001) and a decrease in the percentage of cells in G0/1 (11.8% decrease, *p* < 0.0001) compared to vehicle control (Figure 3B). Importantly, this effect was much smaller than that observed for MIA PaCa-2 cells.

### 2.4. Treatment with Oleuropein and Hydroxytyrosol Promotes Caspase 3/7 Dependent Apoptosis

**Figure 4:**
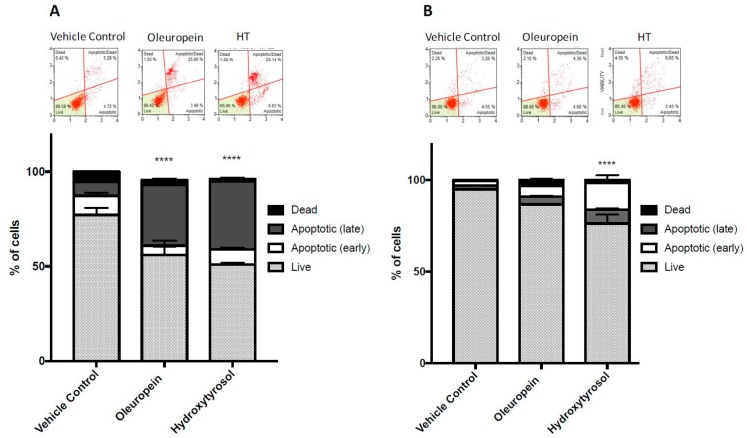
Induction of caspase 3/7-dependent apoptosis of MIA PaCa-2 (**A**) and HPDE (**B**) cells treated with oleuropein (200 µM) and hydroxytyrosol (100 µM) for 48 h. Bar graphs show the percentage of live, early apoptotic, late apoptotic and dead cells determined by analysis of the activation of caspase 3/7. Ordinary two-way ANOVA and Tukey’s multiple comparisons test compare total apoptotic cells in treated cells (oleuropein or hydroxytyrosol) to vehicle control. **** *p* < 0.0001.

Caspase 3 and 7 are activated downstream in the apoptosis cascade and result in the cleavage of protein substrates and the disassembly of the cell [30]. Therefore, the activation of caspase 3/7 measured by fluorescent tagging and subsequent flow cytometry was used to determine the induction of apoptosis. In cells expressing caspase 3/7, the fluorescent dye (MUSE caspase 3/7 reagent) was able to bind to the DNA, while the dead cell marker (7-AAD) entered membrane-compromised, later-stage apoptotic and dead cells. The number of fluorescently labelled cells expressing caspase 3/7 was counted by MUSE flow cytometry. Treatment of MIA PaCa-2 cells with either oleuropein or hydroxytyrosol caused a significant increase in the percentage of cells expressing activated caspase 3/7 (Figure 4A) with the total percentage of cells (early + late apoptosis) increasing from 7.93% (vehicle control) to 40.63% after oleuropein treatment (*p* < 0.0001) and 47.17% after hydroxytyrosol treatment (*p* < 0.0001). The effect on HPDE cells was much smaller, with the total percentage of HPDE cells with caspase 3/7 activation only increasing from 4.6% (vehicle control) to 10% after oleuropein (*p* = 0.613) and 22.01% after hydroxytyrosol (*p* < 0.0001) treatment (Figure 4B).

### 2.5. Differential Expression of Bcl2 Family Proteins Following Treatment with Oleuropein and Hydroxytyrosol

**Figure 5:**
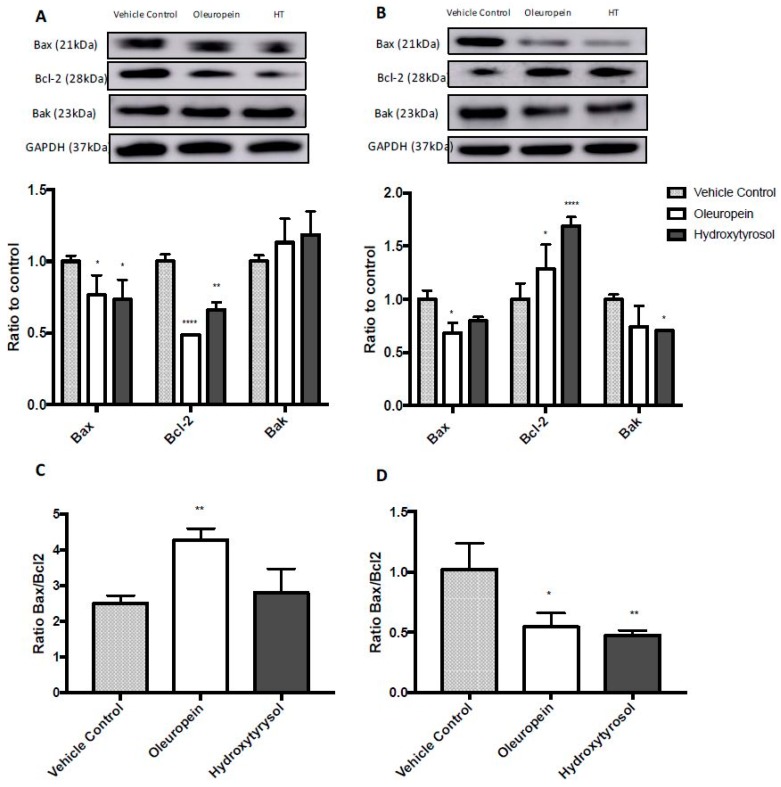
Expression of Bax, Bak and Bcl-2 in MIA PaCa-2 (**A**) and HPDE (**B**) cells treated with oleuropein (200 µM) and hydroxytyrosol (HT) (100 µM) or vehicle control as assessed using gel electrophoresis and Western blotting. GAPDH was used as a loading control. Results displayed as optical density measurements of target antibody/GAPDH/control average; hence, results are represented as fold change. Ratio of the expression of Bax to Bcl-2 in MIA PaCa-2 (**C**) and HPDE (**D**) cells. For (**A**,**B**), ordinary two-way ANOVA with Tukey’s multiple comparisons test compares protein expression of treated cells (oleuropein or hydroxytyrosol) to vehicle control; for (**C**,**D**), ordinary one-way ANOVA with Tukey’s multiple comparisons test compares Bax/Bcl-2 ratio of treated cells to vehicle control. * *p* 0.05, ** *p* 0.01, *** *p* 0.001, **** *p* < 0.0001.

The Bcl-2 family of proteins are involved in the regulation of apoptosis [31]. To determine if Bcl-2 family members were involved in oleuropein-induced apoptosis, the expression of Bax, Bak and Bcl-2 were determined using gel electrophoresis and Western blotting. Results were normalised to GAPDH expression and expressed as fold change compared to vehicle control cells. Interestingly, expression of the pro-apoptotic protein Bax, decreased in MIA PaCa-2 cells (Figure 5A) after oleuropein and hydroxytyrosol treatment (23.4% and 26.6% decrease, *p* = 0.035 and 0.017, respectively). Expression of the anti-apoptotic protein Bcl-2 also decreased (51.4% and 33.7% decrease, *p* < 0.0001 and 0.0027, respectively). However, there was no significant change (Figure 5A) in the expression of Bak (oleuropein *p* = 0.302 and hydroxytyrosol *p* = 0.105). Additionally, oleuropein or hydroxytyrosol treatment of HPDE cells significantly decreased the expression of Bax (31.5% and 20.3% decrease, *p* = 0.016 and 0.013, respectively) and Bak (25.6% and 29.5% decrease, *p* = 0.052 and 0.024, respectively) while increasing the expression of Bcl-2 (28.9% and 69.2% increase, *p* = 0.027 and <0.0001, respectively) (Figure 5B).

A more important indicator of survivability, than the individual expression levels of each apoptosis protein, is the ratio of Bax to Bcl-2 expression. The Bax/Bcl-2 ratio in MIA PaCa-2 cells treated with oleuropein was almost double that of vehicle control cells (control = 2.5, oleuropein = 4.3, *p* = 0.007) (Figure 5C). For hydroxytyrosol-treated cells the Bax/Bcl-2 ratio was not significant different compared to controls (control = 2.5, hydroxytyrosol = 2.7, *p* = 0.72) (Figure 5C). Interestingly, in HPDE cells treated with either oleuropein or hydroxytyrosol, the Bax/Bcl-2 ratio was more than halved (control = 1.02, oleuropein = 0.55, *p* = 0.012 and hydroxytyrosol = 0.47, *p* = 0.0063) (Figure 5D). These data suggest that oleuropein may have induced apoptosis in MIA PaCa-2 cells via the regulation of the expression of mitochondrial proteins while oleuropein and hydroxytyrosol may have had a protective effect on HPDE cells.

### 2.6. Gene and Protein Expression Changes in MIA PaCa-2 Cells Following Treatment with Oleuropein and Hydroxytyrosol

To gain a better understanding of the mechanism by which oleuropein and hydroxytyrosol induce apoptosis in MIA PaCa-2 cells, and the protective role of oleuropein in HPDE cells, the changes in gene expression were determined. An mRNA microarray was utilised to determine the changes in gene expression after treatment with oleuropein or hydroxytyrosol (Table 2). Genes with significant, high fold changes that have a known role in pancreatic cancer were selected for further validation at the protein level by using gel electrophoresis and Western blotting.

**Figure 6:**
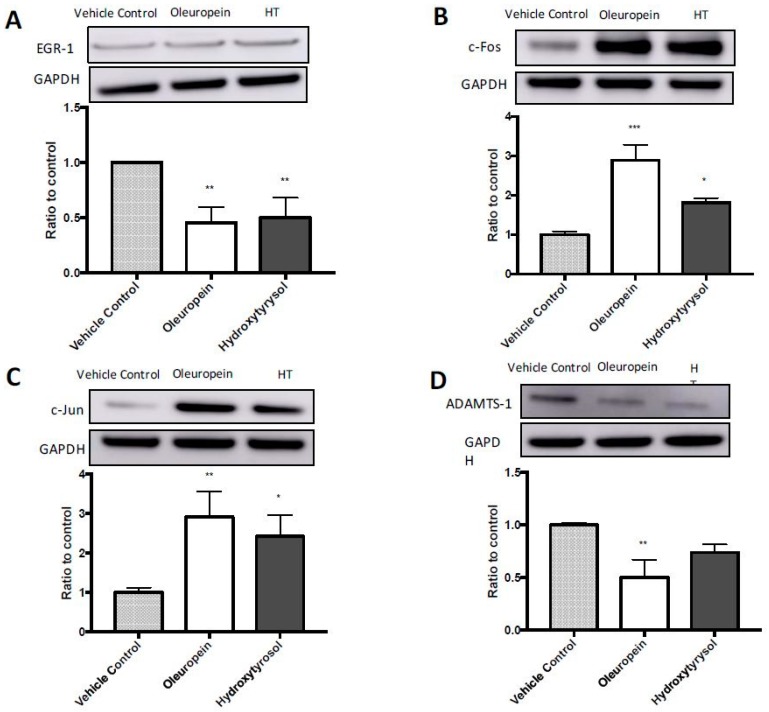
Protein expression of: EGR-1 (**A**); c-Fos (**B**); and c-Jun (**C**) in MIA PaCa-2 cells; and ADAMTS-1 in HPDE cells (**D**). GAPDH was used as a loading control. Results displayed as optical density measurements of target antibody/GAPDH/control average; hence, results are represented as fold change. Ordinary one-way ANOVA with Tukey’s multiple comparisons test compares the expression of protein from treated cells (oleuropein or hydroxytyrosol) to vehicle control. * *p* 0.05, ** *p* 0.01, *** *p* 0.001.

Studies suggest that Early Growth Response-1 (*EGR-1*) is a cancer suppressor gene and accordingly, it was found that *EGR-1* was significantly upregulated in oleuropein (8-fold, *p* = 0.018) and hydroxytyrosol (20-fold, *p* = 0.018) treated MIA PaCa-2 cells (Table 2). Considering *EGR-1* has previously been shown to induce apoptosis in pancreatic cancer [32], *EGR-1* was chosen for further validation at the protein level to determine if this change in gene expression resulted in a functional effect. However, despite the increased expression observed in the microarray (Table 2), this was not reflected at the protein level (Figure 6A). In fact, the protein expression of EGR-1 was significantly reduced after treatment with oleuropein (55% decrease *p* = 0.006) and hydroxytyrosol (50% decrease, *p* = 0.008).

*JUN* and *FOS* are known proto-oncogenes which dimerize to form the transcription factor AP-1. More specifically, forced expression of c-Jun and c-Fos has previously been linked to the induction of apoptosis in pancreatic cancer cells in vitro [33]. In the present study (Table 2), the expression of *JUN* significantly increased 4.6-fold after oleuropein treatment (*p* = 0.000126) and 4.7-fold after hydroxytyrosol treatment (*p* = 0.000041). The expression of *FOS* also increased 2.4-fold after oleuropein treatment (*p* = 0.007736) and 5-fold after hydroxytyrosol treatment (*p* = 0.000103) (Table 2). Due to the relationship between c-Jun and c-Fos, and apoptosis in pancreatic cancer cells, the effects on c-Jun and c-Fos were determined at the protein level. The protein expression of c-Jun increased in both oleuropein (291% increase, *p* = 0.008) and hydroxytyrosol-treated cells (242% increase, *p* = 0.029) (Figure 6C). This trend was also observed with c-Fos; c-Fos protein expression increased in MIA PaCa-2 cells treated with oleuropein (289% increase, *p* = 0.0002) and hydroxytyrosol (182% increase, *p* = 0.015) (Figure 6B).

### 2.7. Reduced Expression of ADAMTS1 in HPDE Cells Following Treatment with Oleuropein

Expression of *ADAMTS1* has been positively correlated with disease progression in cancers of different origins [34]. The gene expression of *ADAMTS1* in HPDE cells after treatment with oleuropein was reduced 2.2-fold (*p* = 0.00003) (Figure 6D). Therefore, to gain an insight into the potential protective role of oleuropein in HPDE cells, *ADAMTS1* was validated at the protein level. The protein expression of ADAMTS1 was also significantly reduced in cells treated with oleuropein (50% decrease, *p* = 0.003) (Figure 6D). However, the effect of hydroxytyrosol on the expression of ADAMTS1 by HPDE cells was not statistically significant (26% decrease, *p* = 0.055) (Figure 6).

## 3. Discussion

The epidemiological link between the Mediterranean diet and pancreatic cancer [29] offers a potential avenue for the exploration of biophenols which may possess anti-pancreatic cancer potential. Oleuropein and hydroxytyrosol are biophenols found in olives, olive leaves and olive oil and their consumption has been linked to health benefits [35,36]. Additionally, the anti-cancer potential of oleuropein and hydroxytyrosol has been described in cancers of several different origins [16,17,37]; however, the present study is the first to investigate their activity in pancreatic cancer.

Oleuropein treatment reduced the viability of pancreatic cancer cells (MIA PaCa-2) but the viability of non-tumorigenic cells (HPDE) was not affected, augmenting oleuropein’s potential as a chemotherapeutic agent for pancreatic cancer. Oleuropein has previously displayed similar effects towards prostate cells by reducing the proliferation of prostate cancer cells while displaying an antioxidant effect on non-malignant prostate cells [18]. The authors attributed this selectivity to the sensitivity of cancer cells to ROS generation. Many human cancer cell types exist in a highly oxidative state compared to their normal tissues and, therefore, the selective activity of oleuropein on MIA PaCa-2 cells could be due to their increased sensitivity towards ROS. Moreover, hydroxytyrosol is a more potent antioxidant than oleuropein [38], potentially explaining the lower IC_50_ of hydroxytyrosol in MIA PaCa-2 cells compared to oleuropein.

Changes in the cell cycle are often an early indicator of cellular stress leading to apoptosis. In the present study, treatment of MIA PaCa-2 cells with oleuropein or hydroxytyrosol caused cell cycle arrest at G2 phase of the cell cycle. Oleuropein has been shown to cause cell cycle arrest in neuroblastoma cells by down-regulation of Cylin-D1/2 and 3, and CDK4 and 6 while up-regulating p53 [39]. Hydroxytyrosol has also previously caused cell cycle arrest in prostate cancer cells by inhibiting cyclin-D1/E and CDK2/4 and inducing inhibitory p21/p27 [40]. Therefore, oleuropein and hydroxytyrosol may be exerting cell cycle arrest via changing the expression of specific cyclins and CDKs involved in G2/M phase. However, this was not investigated in the present study.

Resistance to chemotherapy occurs primarily due to cellular evasion of apoptosis. This evasion can lead to deregulated cell proliferation and subsequent tumour formation. Therefore, the induction of tumour cell apoptosis without displaying toxicity towards surrounding normal cells is an effective chemotherapy strategy [41]. In the present study, the concentrations of oleuropein required to achieve a reduction in viability of pancreatic cancer cells were quite high for a biological context (Table 1). Nevertheless, these data are useful to determine the appropriate dose of oleuropein for in vivo models of pancreatic cancer. Additionally, it is important to understand how drugs behave in vitro as cellular toxicity can arise via several mechanisms. For these reasons, the ability of oleuropein and hydroxytyrosol to induce apoptosis was determined.

The Bcl-2 family of proteins regulate apoptotic mitochondrial events, such as, the ability of ceramide to form channels in the mitochondrial outer membrane. Anti-apoptotic proteins (Bcl-2) inhibit ceramide channels while the pro-apoptotic proteins (Bax and Bak) enhance these channels and lead to the release of pro-apoptotic proteins into the cytosol, which initiates the execution phase of apoptosis [42]. For this reason, the Bax/Bcl-2 ratio is an important indicator of cell survivability. In this study, the treatment of MIA PaCa-2 cells with oleuropein or hydroxytyrosol increased the Bax/Bcl-2 ratio, followed by caspase 3/7-dependent apoptosis. In contrast, Bax/Bcl-2 decreased in HPDE cells after treatment with olive biophenols. These results demonstrate the selective activity of oleuropein and hydroxytyrosol in pancreatic cells.

Olive biophenols have previously been shown to induce apoptosis in cancer cells. Oleuropein treatment has caused an increase in the Bax/Bcl-2 ratio in lung cancer cells (A549) [43], breast cancer cells [44] and neuroblastoma cells (SH-SY5Y) [39], in each case activating the caspase cascade causing cells to undergo apoptosis. Similarly, hydroxytyrosol has previously increased the Bax/Bcl-2 ratio in prostate cancer cells [40]. The previous studies credit varying upstream mechanisms for the change in expression of Bcl-2 family proteins, which appear to be cell type specific.

To elucidate the mechanisms underlying the selective activity of oleuropein and hydroxytyrosol, changes in gene expression after treatment were assessed using microarrays. Major genes differentially expressed in MIA PaCa-2 cells after treatment include *EGR-1*, *JUN* and *FOS*. *EGR-1* has been linked with apoptosis in MIA PaCa-2 cells treated with vitamin E δ-tocotrienol by causing an increase in the Bax/Bcl-2 ratio and subsequent caspase cascade inducing apoptosis [32]. In the present study, oleuropein and hydroxytyrosol treatment also increased the gene expression of *EGR-1* and the Bax/Bcl-2 ratio as well as caspase 3/7 activation in MIA PaCa-2 cells. However, EGR-1 protein expression decreased as a result of treatment with oleuropein and hydroxytyrosol; therefore, the role of *EGR-1* in oleuropein and hydroxytyrosol-induced apoptosis of MIA PaCa-2 cells is still to be determined.

The proteins c-Jun and c-Fos dimerize to form Activator Protein-1 (AP-1). The AP-1 site is a ubiquitous regulatory element that is found in a wide range of promoter and enhancer regions. They bind to AP-1 DNA recognition elements and control cell proliferation, transformation, survival and death. The functions of Fos-Jun family proteins depend on the specific cell type in which they are expressed [45,46]. We found that oleuropein and hydroxytyrosol treatment of MIA PaCa-2 cells caused an increase in the gene expression of *JUN* and *FOS* and an increase in the protein expression of c-Jun and c-Fos. Previously, the forced expression of c-Jun and c-Fos has induced apoptosis in neuronal cells [47,48]. Additionally, treatment of myelodysplastic cells with the plant-derived compound, withaferin A, caused an increase in the mRNA expression of *JUN* and *FOS* and their subsequent protein expression and resulted in downstream activation of apoptosis [49]. More specifically, Ren et al. [33] were able to show that increasing the expression of c-Jun and c-Fos in MIA PaCa-2 cells caused the downstream induction of apoptosis via activation of Bim and the subsequent effect on Bcl-2 family proteins. These previous studies support the theory that the induction of apoptosis in this study could be due to an increased expression of c-Jun and c-Fos, which dimerize into AP-1 and result in activation of the AP-1/JNK pathway. However, more work is needed to substantiate this hypothesis.

The effects observed in MIA PaCa-2 cells in the present study and the previous studies showcase the ability of oleuropein and hydroxytyrosol to induce apoptosis via several different pathways. However, in this study, oleuropein and hydroxytyrosol decreased the Bax/Bcl-2 ratio in HPDE cells, suggesting a protective effect on these non-tumorigenic cells. In addition to their potent cytotoxic activities previously described, these olive biophenols have also displayed cytoprotective effects. Kalaiselvan et al. [50] found that oleuropein, hydroxytyrosol and even tyrosol exhibited a protective effect on rat liver cells exposed to the harsh environmental toxin, 2,3,7,8-tetrachlorodibenzo-P-dioxin (TCDD) by decreasing the Bax/Bcl-2 ratio. Additionally, olive oil treatment of hippocampus CA1 neurons following ischemia in mice also reduced apoptosis by decreasing Bax and increasing Bcl-2 expression [51]. The cytoprotective activity of oleuropein was also observed by Geyikoglu et al. [52], who found that the administration of 200 mg/kg/day of oleuropein for 3 days modulated oxidative stress and completely reversed 8-OHdg production in mouse kidneys after treatment with the chemotherapy drug, cisplatin. These studies support the observed protective effects of oleuropein and hydroxytyrosol in HPDE cells.

*ADAMTS1* is a metalloprotease that remodels the ECM (extracellular matrix) through the proteolytic degradation of key substrates such as collagen [53]. Low *ADAMTS1* expression has been linked to tumorigenesis in some cancers. However, the specific role of *ADAMTS1* in pancreatic cancer is yet to be determined. In the present study, lower levels of *ADAMTS1* were expressed in oleuropein and hydroxytyrosol-treated HPDE cells than in control cells. Low expression of *ADAMTS1* in pancreatic cancer tissue compared to normal pancreas tissue has previously been studied [34]; however, large variability in the data resulted in the relationship being non-significant (*p* = 0.206). Despite this, low *ADAMTS1* expression in primary tumours compared to normal tissue is a common trend that can be correlated with disease progression. Reports on prostate [54], colon [55] and lung cancer [56] have shown lower expression of *ADAMTS1* in primary cancers compared to non-tumorigenic tissue. Further investigations have revealed that epigenetic silencing through promoter hyper-methylation to be the key mechanism underlying *ADAMTS1* suppression during tumour development. Therefore, the methylation state of *ADAMTS1* has been proposed as a potential early biomarker for colon, prostate and non-small cell lung cancer [56]. As mentioned, oleuropein treatment of the non-tumorigenic pancreas cells (HPDE) decreased the expression of *ADAMTS1* in the present study; this result conflicts the protective activity exhibited by oleuropein. Moreover, considering the previous reports of *ADAMTS1* and disease progression, it is possible that the observed protective effects of oleuropein on HPDE cells could instead be the first stages of cellular transformation leading to tumorigenesis. However, more research is needed to determine the role of oleuropein and hydroxytyrosol on *ADAMTS1* functions as well as the role of *ADAMTS1* on pancreatic cells and pancreatic cancer disease progression. Due to the complex nature of tumorigenesis, these data highlight the importance of determining chemo-preventative mechanisms.

For the first time, anti-pancreatic cancer properties of oleuropein and hydroxytyrosol have been determined in vitro. Oleuropein and hydroxytyrosol arrested the cell cycle, increased the Bax/Bcl-2 ratio, increased activation of caspase 3/7 and induced apoptosis in pancreatic cancer cells (MIA PaCa-2). Increased expression of c-Jun and c-Fos was also observed in oleuropein and hydroxytyrosol-treated cells and, therefore, dimerization of c-Jun and c-Fos into AP1 is a potential underlying mechanism for oleuropein and hydroxytyrosol-induced apoptosis in MIA PaCa-2 cells. However, more work is needed to validate these findings. Additionally, oleuropein did not display toxicity towards non-tumorigenic cells (HPDE); in fact, a putative protective effect was observed. However, the downregulation of *ADAMTS1* in oleuropein treated cells conflicts its protective label. Therefore, more work is also needed to determine if the observed protective effects of oleuropein on non-tumorigenic pancreas cells could lead to cancer prevention.

## 4. Materials and Methods

### 4.1. Materials

Oleuropein, hydroxytyrosol, tyrosol, isopropranol, glycogen, ethanol, 2-mercaptoethanol, and Roche cOmplete protease inhibitor cocktail as well as the reagents for the RIPA lysis buffer (made from 150 mM NaCl, 1.0% IGEPAL CA-630, 0.5% sodium deoxycholate, 0.1% sodium dodecyl sulphate (SDS) and 50 mM Tris-HCl, pH 8.0) were purchased from Sigma Aldrich (Temecula, MS, USA). CCK-8 reagent was purchased from Dojindo Molecular Technologies Inc., (Rockville, MD, USA). Dulbecco’s Modified Eagle Medium: Nutrient Mixture F-12 (DMEM-F12), Keratinocyte Serum-Free Media (K-SFM), Roswell Park Memorial Institute medium (RPMI), Iscove’s Modified Dulbecco’s Medium (IMDM), trypsin-EDTA, l-glutamine, phosphate buffered saline (PBS) and TRIzol reagent were purchased from Invitrogen (Carlsbad, CA, USA). Foetal Bovine Serum (FBS) was purchased from Interpath (Heildberg West, Australia). Luminata Classico western horse radish peroxidase substrate and MUSE™ cell cycle and caspase 3/7 reagent kits were purchased from MERK-Millipore (Temecula, MA, USA). Gene Chip WT PLUS reagent kits were purchased from Affymetrix (Carlsbad, CA, USA). RIPA lysis buffer and bicinchoninic acid (BCA) protein assay kit was purchased from Thermofisher (Mulgrave, Australia). Pancreatic cancer cells, MIA PaCa-2, CFPAC-1, BxPC-3 and ASPC-1, were purchased from ATCC (Manassas, VA, USA) and immortalised normal pancreatic ductal epithelial cells (HPDE) were a gift from the lab of Dr. M. Tsao (MD, FRCPC, University of Health Network, Toronto, ON, Canada). All cell lines were authenticated by CellBank Australia (Westmead, Australia).

### 4.2. Pancreas Cell Culture

Human pancreatic cancer cells, MIA PaCa2, were cultured in DMEM-F12, supplemented with 10% FBS, 2.5% horse serum and l-glutamine (100 µg/mL). BxPC-3 and ASPC-1 were cultured in RPMI supplemented with 10% FBS and l-glutamine (100 µg/mL). CFPAC-1 cells were cultured in IMDM supplemented with 10% FBS and l-glutamine (100 µg/mL). Human Pancreas (HPDE) cells were cultured in K-SFM supplemented with BPE and EGF. All cells were grown and maintained at 37 °C under 5% CO_2_.

### 4.3. Assessment of Cell Growth Inhibition

Cell growth inhibition was determined using the Dojindo Cell Counting Kit-8. Cells were seeded into a 96 well plate at 3000–10,000 cells per well and allowed to adhere for 24 h. Cells were then treated within the range of 0–300 μM of each compound, 0–50 nM gemcitabine (positive control) or 0.5% DMSO (vehicle control). After 72 h, 10% CCK-8 solution in media was added before incubating at 37 °C for 180 min. The absorbance was measured at 450 nm and cell growth inhibition was determined as the IC_50_. All experiments were performed in triplicate. 

### 4.4. Apoptosis Assay

The induction of apoptosis was evaluated by assessing caspase 3/7 activation. MIA PaCa-2, and HPDE cells were seeded into 12 well plates at 30,000 and 100,000 cells/well, respectively. After 24 h, cells were treated with oleuropein (200 μM) or hydroxytyrosol (100 μM) for 48 h before washing with PBS and dislodging with 0.25% trypsin EDTA. Cells were diluted to 300 cells/μL prior to staining. MUSE caspase 3/7 reagent working solution was prepared by diluting the stock solution with 1× PBS. The MUSE Caspase 7-AAD reagent was diluted with 1× assay Buffer at a ratio of 1:74. Cells were stained by adding 5 μL of the caspase 3/7 working solution to 50 μL of diluted cells and incubating for 30 min at 37 °C and 5% CO_2_ before adding 150 μL of Caspase 7-AAD working solution and incubating for 5 mins at room temperature. All assays were performed in triplicate. Fluorescence was read on a MUSE cell analyser (MERCK Millipore, Sydney, Australia). Results were expressed as percentage of live cells, early and late apoptotic cells as well as percentage of dead cells.

### 4.5. Cell Cycle Analysis

The effect of oleuropein on the cell cycle was analysed by DNA staining with propidium iodide (MERCK Millipore cell cycle kit). MIA PaCa-2 and HPDE cells were seeded into 12 well plates at 30,000 and 100,000 cells/well, respectively. After 24 h, cells were treated with oleuropein (200 μM) or hydroxytyrosol (100 μM) for 24 h before washing with PBS and dislodging with 0.25% trypsin EDTA. Cells were washed with PBS before fixing in ice cold 70% ethanol and storing at −20 °C for a minimum of 3 h prior to staining. For staining, cells were centrifuged at 300 g for 5 min, washed in PBS before resuspending in 200 µL of cell cycle reagent and incubated for 30 min at ambient temperature in the dark. Fluorescence was read on a MUSE flow cell analyser and DNA content profile histograms were produced. Results were expressed as percentage of cells in G0/1, S and G2/M phases of the cell cycle. All assays were performed in triplicate.

### 4.6. Assessment of the Effect of Oleuropein and Hydroxytyrosol on Gene Expression

HPDE and MIA PaCa-2 cells were seeded into 12 well plates at a concentration of 100,000 or 30,000 cells/well, respectively, and allowed to adhere for 24 h before treatment with either oleuropein (200 µM), hydroxytyrosol (100 µM) or vehicle control for 24 h. Total RNA was extracted after 18 h using TRIZOL reagent (Invitrogen, Carlsbad, CA, USA) as per the manufacture’s guidelines, with an overnight −30 °C isopropanol and glycogen precipitation. The quantity and integrity of the RNA preparation was analysed using a 6000 Nano Kit on an Agilent 2100 Bioanalyzer (Agilent Technologies, Santa Clara, CA, USA). RNA integrity number (RIN) integrity scores were all over 7. Total RNA (100 ng) was used as starting material. The WT PLUS amplification and labelling process was prepared according to Affymetrix protocols. Fragmented and biotinylated ss-cDNA preparations (5.5 μg) were hybridized to HTA 2.0 Arrays, which were subsequently washed, stained, and scanned using a GeneChip Fluidics station and GeneChip Scanner 3000 (Affymetrix, Carlsbad, CA, USA) according to the Affymetrix protocol. Array images were processed using the Affymetrix GeneChip command console (AGCC) to produce probe intensity data (*cel* files). After using the Expression console to normalise and examine data quality, only the microarrays meeting acceptable Affymetrix quality control criteria were considered for further analysis. The Expression console was then used to create probe level summarisation files (CHP) from the *cel* files. The Transcriptome analysis console (TAC) was used to convert the CHP files into a visual representation of the differentially expressed genes. Statistical analysis was preformed using One-Way ANOVA (unpaired) on genes displaying a significant fold change [(linear) < −2 or >2, and *p*-value < 0.05)].

### 4.7. Protein Expression

HPDE and MIA PaCa-2 cells were seeded into 12 well plates at a concentration of 100,000 or 30,000 cells/well, respectively, and allowed to adhere for 24 h before treatment with either oleuropein (200 µM), hydroxytyrosol (100 µM) or vehicle control (0.5% DMSO) for 24 h. Whole cell lysates were collected by lysis with RIPA lysis buffer containing protease inhibitor for 30 min and subsequent centrifugation at 10,000× *g* at 4 °C for 30 min. Protein concentration was determined by a micro BCA protein assay kit according to the manufacturer’s instructions (Thermofisher, Mulgrave, Australia). Lysates were dissolved in sample buffer (0.35 M Tris-HCl pH 6.8, 30% glycerol, 10% SDS and bromophenol blue) and reducing agent (9% 2-mercaptoethanol) before heating at 75 °C for 10 min. Samples were separated on NuPAGE^®^ Novex^®^ Midi Bis-Tris 4–12% gels followed by transfer onto nitrocellulose membrane (GE Healthcare, Sydney, Australia). The membranes were blocked using 10% skim milk in TBST, washed with TBST and probed with primary antibodies for 2 h and secondary antibodies for 1 h before addition of a chemiluminescent substrate (Luminata Classico western Horse Radish Peroxidase substrate, MERCK-Millipore, Temecula, MA, USA). Bands were revealed using an Amersham Imager 600 (GE Healthcare, Sydney, Australia). The following primary antibodies were used: rabbit polyclonal anti-Bax (dilution 0.125 µg/mL; abc11; MERK Millipore, Burlington, MA, USA), rabbit monoclonal anti-Bak (dilution 1:1000; 06-536; MERK Millipore), mouse monoclonal anti-Bcl-2 (dilution 2 µg/mL; 05-729: MERK Millipore), rabbit polyclonal anti-ADAMTS1 antibody (dilution 1 µg/mL; ab113847; abcam), rabbit polyclonal anti-c-Fos antibody (dilution 1:500; ab209795; abcam), rabbit monoclonal anti-c-Jun antibody (dilution 1:2000; (E254) ab32137; abcam), rabbit polyclonal anti-EGR-1 antibody (dilution 1:500; ab208780; abcam), rabbit polyclonal anti-GAPDH (dilution 1:1000; 00-18231; Biovision, San Fransisco, CA, USA). The secondary antibodies were rabbit anti-goat (dilution 1:5000; 172-1034; BioRad, Hercules, CA, USA), mouse anti-goat (dilution 1:5000; 170-6516; BioRad).

### 4.8. Statistics

GraphPad Prism Version 7.0 was used to determine the IC_50_ of normalised and transformed viability data. Ordinary two-way ANOVA followed by Tukey’s multiple comparisons test was conducted on cell cycle, caspase 3/7 expression and expression of apoptosis proteins data to compare treated versus vehicle control cells. Ordinary one-way ANOVA followed by Tukey’s multiple comparisons test was conducted on Bax/Bcl-2 ratio and gene expression data to compare treated versus vehicle control cells. Significance was set at *p* < 0.05.

## Figures and Tables

**Table 1:** Viability of pancreatic cancer cells (MIA PaCa-2, BxPC-3, CFPAC-1 and ASPC-1) and non-tumorigenic pancreas cells (HPDE) when treated with 0–300 µM of oleuropein, hydroxytyrosol or tyrosol. Values represent concentration required to achieve a 50% reduction in viability (IC_50_).

Cell Line	Oleuropein (µM)	Hydroxytyrosol (µM)	Tyrosol (µM)	Gemcitabine (nM)
MIA PaCa-2	150.1	75.1	>300	31.02
BxPC-3	>300	>300	>300	3.6
CFPAC-1	>300	>300	>300	2.6
ASPC-1	>300	>300	>300	12
HPDE	>300	65.5	>300	0.04

**Table 2:** Fold change in the gene expression of *JUN*, *FOS* and *EGR-1* in pancreatic cancer cells (MIA PaCa-2) and *ADAMTS1* in non-tumorigenic cells (HPDE) when treated with oleuropein or hydroxytyrosol.

Cell Line	Gene Symbol	Treatment	Fold Change (Linear) (VS. Control)	ANOVA *p*-Value (VS. Control)
MIA PaCa-2	JUN	Oleuropein	4.64	0.000126
		Hydroxytyrosol	4.68	0.000041
	FOS	Oleuropein	2.41	0.007736
		Hydroxytyrosol	4.98	0.000103
	EGR-1	Oleuropein	8.01	0.00083
		Hydroxytyrosol	20.75	0.000019
HPDE	ADAMTS1	Oleuropein	−2.19	0.00003
		Hydroxytyrosol	-	-
